# Credibility Elements of eWOM Messages in the Context of Health Care Services. A Romanian Perspective

**Published:** 2013-09-25

**Authors:** VL Purcarea, IR Gheorghe, CM Petrescu

**Affiliations:** * “Carol Davila" University of Medicine and Pharmacy, Bucharest; ** National School of Political Studies and Public Administration, “Carol Davila" University of Medicine and Pharmacy, Bucharest

**Keywords:** health care services, electronic word of mouth, argument quality, source credibility, virtual communities

## Abstract

The purpose of this paper is to investigate the Romanian consumers’ determinants of eWOM messages’ perceived credibility in the context of health care services. We selected a sample of 127 women and we administered a questionnaire. We used the partial least squares to uncover the established relationships between the variables of the model, namely the argument strength and the source credibility of a eWOM message and the intention to purchase a health care service based on the information embedded in the eWOM messages. The results revealed that all variables had positive direct correlations with each other but the argument strength of a message has the highest impact on the intention.

## Introduction

Along with the development of technology, the internet has begun to penetrate the people’s everyday lives. Therefore, consumers are increasingly using more and more the computer-mediated communication in order to share ideas, experiences, ask for advice and build communities [**1**]. The online communities are “aggregations that emerge from the internet when enough people carry on those public discussions long enough, with sufficient human feeling, to form webs of personal relationships in cyberspace" [**2**]. Moreover, it was argued that a virtual community is a “technology supported cyberspace, centered upon communication and interaction of participants, resulting in a relationship being built up". Internet users seek and read other consumers’ recommendations through different instruments of these social interactive groups, which facilitate the interaction between members. As such, the oldest and richest form of online user interaction is the discussion forum [**4,5**]. The discussion forum is an online type of “town forum" or “virtual meeting place" which is centered on debates around a product, a service or a lifestyle [**1,6**]. Usually, a virtual community works as a reference group being linked to the Electronic Word-of-Mouth (eWOM), the new consumer sharing activity [**7**]. More specifically, eWOM is any “positive or negative statement made by potential, actual or former customers about a product or a company, which is made available to a multitude of people and institutions via the internet" [**7**]. 

 Nowadays, in the light of growth rate of the internet usage, eWOM had a massive impact on the people’s behaviors and decisions [**8**]. Furthermore, current studies on eWOM focused mainly on the effectiveness of eWOM communication [**9**], variables specific for the outcomes of eWOM related to both receivers’ psychological states such as attitude [**8,10**], information adoption [**6,10,11**], purchase intention [**12,13**], usefulness of information [**14**], and, related to the communicators of the eWOM messages such as motivations [**6,15**] and their characteristics such as attractiveness [**16**]; homophily [**17**], as well as on the eWOM messages’ particularities [**9,13,18**] explored in different contexts. However, research on eWOM credibility and, implicitly, its characteristics has been somewhat neglected in literature or defined too broad. 

 eWOM credibility is defined as the extent to which one person perceives the eWOM recommendation as believable, true or factual [**19,20**]. It is worth mentioning that credibility suggested by eWOM refers to the online message itself as well as to the source, meaning the communicator of the message. Therefore, if individuals consider the incoming information credible, they will have more confidence in adopting the eWOM messages and use them for making decisions [**21**]. Still, it was concluded that a key factor in the information process is the receiver’s judgment of the information credibility [**22**]. 

 The information adoption of a eWOM message refers to an individual’s assimilation of knowledge comprised in the forum posts, who, consequently, will give meaning to the transferred information [**23**]. In literature, there are several theories, which are used to explain how people judge when they adopt an idea, knowledge or information contained in a eWOM message. To exemplify, the vast majority of online consumers unconsciously evaluate the eWOM messages by applying one of the following theories: 

 - The Elaboration Likelihood Model (ELM) which refers mainly to the consumer-information process, which consists of two routes: the central route and the peripheral route [**24**]. When people take a central route they usually look carefully at the relevant information before forming an attitude whereas when they take the peripheral route they tend to adopt mental shortcuts that require an effort that is less cognitive. 

 - The Heuristic-Systematic Model (HSM) encompasses two methods of processing the information, namely, systematic and heuristic. The systematic processing involves “a much more comprehensive effort to analyze and understand information" [**25**], while the heuristic processing refers to a “less cognitive effort" and is adopted when individuals lack motivation, time and knowledge [**26,27**]. 

 - The Attribution Theory reflects certain positions adopted by individuals when they read eWOM messages, recommend products or complain [**28**]. 

 Reference [**21**] applied the ELM in a theoretical model of information adoption. As such, the central route reflects the nature of arguments in the message while the peripheral route refers to issues and themes that are not directly related to the subject of the message [**24**]. In other words, in the context of computer-mediated communication, the central route is related to the argument quality whereas the peripheral route refers to the source credibility [**21**]. Furthermore, the importance of both information quality and source credibility has been highlighted and strongly validated in prior research [**29,30**]. Most of the times a confusion occurs between the credibility given by a source and the credibility of a message. Thus, to avoid the confusion, source credibility is given by the source’s characteristics while the credibility of a message refers to the message judgment [**31**]. However, there is a direct correlation between the two concepts - that is- the more credible a source is perceived, the more likely the recipient will adopt the eWOM message [**32**].

### Background- Health Care Services

 Health care is a service that most people need but do not want [**33**]. Even if health care services are similar to other services, they have several dissimilar characteristics such as consumers are sick, reluctant, relinquish their privacy and perceive higher risks when a health care service is provided [**33**]. 

 Along with the development of computer-mediated communication (CMC) and the internet, a new typology of consumer appeared, namely the e-patient. The e-patient uses the online environment to search for health information. Consequently, the internet became the harbinger of the consumer movement in health care [**34**], leading to a change in the conventional service encounter along with the patients’ access to virtual communities, known as patients’ online communities (POC) [**35**]. POCs offer health care consumers both technical and practical information in the form of “virtual" second opinions as well as emotional support [**36**]. Moreover, POCs are “communities of unintended interest" [**37**] in which patients post unintended eWOM [**38**]. 

 The past literature showed that informational cues are important for the readers of eWOM messages because they influence their evaluation of the effectiveness of the incoming information. In addition, research which focused on eWOM analyzed the concept applied in different contexts but not health care. Therefore, the purpose of this paper is to investigate the extent to which the eWOM readers of messages on a health care forum in Romania are willing to adopt the online posts as well as uncover which are the factors that favor the adoption. Therefore, in this study we focus on the empirical examination of the Romanian consumers’ determinants of eWOM messages’ perceived credibility in the context of health care services. We attempt to identify and enhance the underlying key determinants related to eWOM messages with the help of a conceptual framework.

## Material and Methods

 In a CMC, the adoption of information, namely the intention to purchase a product or a service by a receiver is influenced by the level of quality of information posted in the message [**11**]. The eWOM message expresses positive, negative statements or a combination of both. Our conceptual framework followed the Information Adoption Model, which studied the correlations established between the characteristics of the message argument quality, source credibility, information usefulness and information adoption in a CMC [**21**]. However, we integrated only argument quality and source credibility in our model because they are the pre-requirements of individuals to accept information [**39**]. In other words, an individual has to understand the message and afterwards judge the source’s credibility. 

 In order to fulfil our objectives we employed a random sampling during July 2012-August 2012. Consequently, our sample was made up of 127 women. We targeted only women because they are the biggest “health care spenders" [**40**] and only forums dedicated to women follow the characteristics suggested by Reference [**5**]. 

 We selected the self-administered questionnaire for the data collection. The questionnaire comprised two parts as it follows: 

a) The first part enhanced the demographic profile of the respondents; 

b) The second part reflected our conceptual framework. We selected some eWOM messages from a forum dedicated to women’s problems and we administered some statements, some were meant to measure the argument quality, some the source’s credibility and some the interaction to purchase a health care consultation based on the eWOM messages on a 5-point Likert scale which ranged from 1- totally disagree to 5-totally agree. 

 Argument quality refers to the persuasive strength of the arguments embedded in an information eWOM message [**41**]. Therefore, we integrated only the argument strength of a message in our model because if the consumers perceive the information in the eWOM message as meeting their needs and requirements, they would adopt the information [**42**]. Basically the evaluation is made, on one hand, in terms of accuracy, format and timeliness of the message [**43**], in terms of accuracy, relevance, understandability, completeness, currency, dynamism, personalization and the variety of the message [**44**] and, on the other hand, in terms of understandability, reliability and usefulness of information [**45**]. Thus, we selected from the Reference [**46**]’s scale which was meant to measure the relevance of a message two statements whereas from the Reference [**47**]’s scale which was meant to measure accuracy, two statements as is illustrated in the annex. 

 A source is thought to be credible when he/she has favourable credentials [**31**]. However, since eWOM communication on a forum is anonymous, the reader of the message has to evaluate a source’s credibility after his/her perceived intention [**48**]. Therefore, a message sent from an unknown source might be perceived as credible if the source gives hints of not benefiting in any way from the message communicated. In our model, we adopted the idea that a receiver of a eWOM message usually evaluates a source’s credibility after his/her trustworthiness [**11,49**]. 

 If a user looks for information about a service, the buying outcome would be evaluated as intention. In our model we applied the Reference [**50**]’s perspective of expressing an intention. As such, they considered an intention as plan, as expectation and as want. 

**Hypothesis **

 a) There is a positive direct correlation between the argument strength and source credibility of a health care eWOM message. 

 b) There is a positive direct correlation between the argument strength and the intention to purchase a health care consultation based on a eWOM message. 

 c) There is a positive direct correlation between a source’s credibility and the intention to purchase a health care consultation based on a eWOM message. 

 We used SPSS version 20 for data collection and WrapPLS version 3.0 to check the validity of the model. The research model was tested by using Partial Least Squares (PLS), a modelling technique that is used in highly complex models [**51**]. 

**Findings**

 The Demographic Profile of Respondents 

 Most women had the ages between 25 and 34 (74.80%), had university degrees (66.10%) and were single (61.40%). Moreover, the vast majority of the participants had professional jobs (22.8%) and their monthly incomes between 1100 and 1500 RON (Romanian Coins) (26.80%). 

**The Conceptual Framework**

 In this section, we will first examine the internal consistency and validity of the model and then assess the structural model. Therefore, the method used for verifying the internal consistency of the model is the Cronbach’s alpha coefficient [**52**]. The Cronbach’s alpha value for the source credibility construct was 0.88, for the argument strength was 0.89, while for the intention to purchase the health care service was 0.95. Following the recommendations of several researchers [**52**] we may conclude that our model has internal consistency. Using the WrapPLS we measured the reliability of the scales included in the model with the help of both composite reliability coefficients (CR) and the average extracted value (AVE) [**53**]. In our case, the CR value for source credibility, argument strength and intention to purchase were 0.94, 0.92 and 0.96, whereas the AVE for the construct was 0.89, 0.76 and 0.90, respectively. 

**Validity of the model**

 A validity of a model does not necessarily mean only the measurement of the theoretical robustness but also the measurement of the relationships established between the variables of the model [**54**]. There are two types of methods in WrapPLS that could test the validity of a model, convergent validity and the discriminant validity [**55**]. There are two conditions which should be met in order for a model to have convergent validity: the p values associated with the loadings to be lower than 0.05 and loadings to be equal or higher than 0.5 [**55**]. From **Table 1** it might be seen that the model has convergent validity.

**Table 1:**
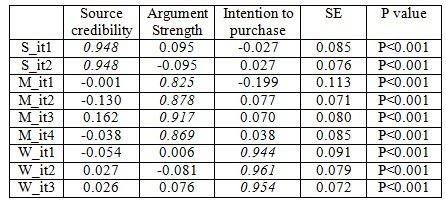
Structure Matrix with Loadings and Cross-loadings of the variables

Discriminant validity is the extent to which the measurement is not a reflection of other variables. Discriminant validity can be demonstrated when the squared root of the AVE for each construct is higher than the correlations between it and the other constructs. **Table 2** shows that the model has discriminant validity [**56**]. 

**Table 2:**
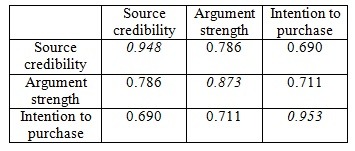
Latent variable correlations and square root of the AVE

**The fitness of the model**

 The fitness of a PLS-based model is measured with the help of three indices: the average path coefficient (APC), the average-R squared (ARS) and the average variance inflation factor (AVIF). In order to access the goodness of fit, the APC and ARS indices should have p values lower than 0.05 and AVIF should be lower than 5 [**55**]. In our case, the APC value is 0.533 at a p level lower than 0.001, ARS value of 0.615 at a p value lower than 0.001 whereas the AVIF value is 3.391 which is lower than 5.

**The structural model**

**Fig. 1:**
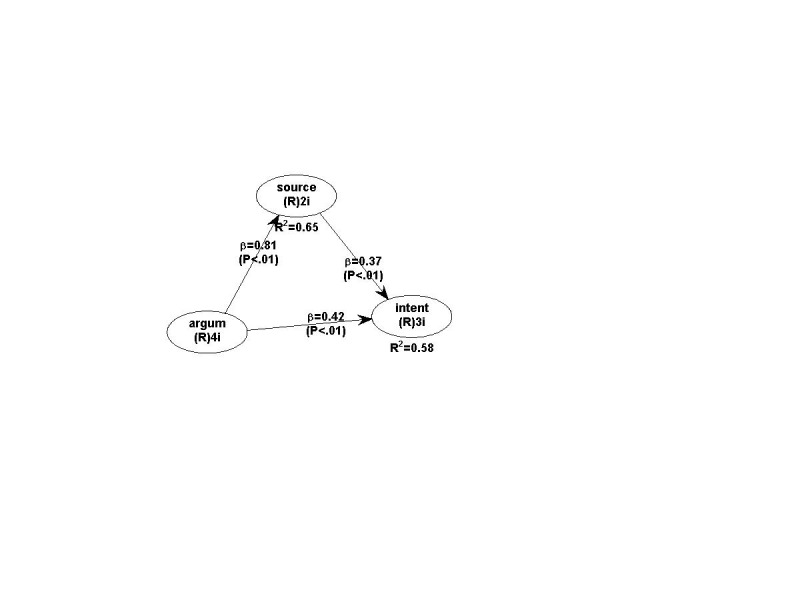
The SEM model

 The PLS-structural equation modelling is based on the partial least squares method and measures the connection between latent variables using beta standardized coefficients and R square values. Our PLS-based SEM model, the path coefficients and their associated p values are illustrated in Figure 1.

The results illustrate that the exogenous variable explains 58% of the variation in the intention to purchase. All the structural paths were found statistically significant in the research model. The argument strength brings a more significant impact over the intention. 

## Discussion

In this study we investigated the drivers that influence a reader adopt information from an eWOM message posted on a forum. We have uncovered that even in health care services the argument strength has the strongest and significant impact on a consumer’s decision. Both the source credibility and argument strength variables explain 58% of the variance, as the rest is explained by other variables. Therefore, before interpreting the results of this study, specialists should pay attention to a number of limits as it follows:

 - As mentioned earlier, the model was extremely simplified in order to enhance the essence of the underlying elements of an eWOM communication;

- Even if the two constructs from the model, explained 58% of the variance of the intention actually it points out that the other important variables might be missing;

 - The other variables of the argument strength should be included in the model such as timeliness or persuasiveness [**41**];

- The other variables of the source’s credibility should be included in the model, such as the source’s expertise [**11**];

 - The results of the study should be extended to the other types of online communities and investigate whether they fit or not;

 - The sample size is relatively small. A larger sample of respondents would have helped in determining a more accurate measurement;

 - The sample should have been made up of a more diverse sample of potential users with other geographic dispersion, professional backgrounds or different health care issues.
